# Progesterone-associated arginine decline at luteal phase of menstrual cycle and associations with related amino acids and nuclear factor kB activation

**DOI:** 10.1371/journal.pone.0200489

**Published:** 2018-07-10

**Authors:** Gernot Faustmann, Andreas Meinitzer, Christoph Magnes, Beate Tiran, Barbara Obermayer-Pietsch, Hans-Jürgen Gruber, Josep Ribalta, Edmond Rock, Johannes M. Roob, Brigitte M. Winklhofer-Roob

**Affiliations:** 1 Human Nutrition & Metabolism Research and Training Center, Institute of Molecular Biosciences, Karl-Franzens University, Graz, Austria; 2 Clinical Division of Nephrology, Department of Internal Medicine, Medical University, Graz, Austria; 3 Clinical Institute of Medical and Chemical Laboratory Diagnostics, Medical University, Graz, Austria; 4 Institute for Biomedicine and Health Sciences, HEALTH, Joanneum Research Forschungsgesellschaft m.b.H., Graz, Austria; 5 Division of Endocrinology, Department of Internal Medicine, Medical University, Graz, Austria; 6 Unitat de Recerca de Lipids I Arteriosclerosi, Facultat de Medicina, Universitat Rovira I Virgili, Facultat Medicina i Ciències de la Salut, Reus, Spain; 7 Unité de Nutrition Humaine, Centre Auvergne Rhône-Alpes, Institut National de la Recherche Agronomique, Saint-Gènes-Champanelle, France; National Institute for Agronomic Research, FRANCE

## Abstract

**Background/Objectives:**

Given their role in female reproduction, the effects of progesterone on arginine and related amino acids, polyamines and NF-κB p65 activation were studied across the menstrual cycle.

**Methods:**

Arginine, ornithine and citrulline as well as putrescine, spermidine, spermine, and N-acetyl-putrescine were determined in plasma, NF-κB p65 activation in peripheral blood mononuclear cells and progesterone in serum of 28 women at early (T1) and late follicular (T2) and mid (T3) and late (T4) luteal phase.

**Results:**

Arginine and related amino acids declined from T1 and T2 to T3 and T4, while progesterone increased. At T3, arginine, ornithine, and citrulline were inversely related with progesterone. Changes (ΔT3-T2) in arginine, ornithine, and citrulline were inversely related with changes (ΔT3-T2) in progesterone. Ornithine and citrulline were positively related with arginine, as were changes (ΔT3-T2) in ornithine and citrulline with changes (ΔT3-T2) in arginine. At T2, NF-κB p65 activation was positively related with arginine. Polyamines did not change and were not related to progesterone. All results described were significant at *P* < 0.001.

**Conclusions:**

This study for the first time provides data, at the plasma and PBMC level, supporting a proposed regulatory node of arginine and related amino acids, progesterone and NF-κB p65 at luteal phase of the menstrual cycle, aimed at successful preparation of pregnancy.

## Introduction

The menstrual cycle is characterized not only by a strong increase between follicular and luteal phase in basal and resting metabolic rate [1,2], but also in amino acid oxidation [2] and nitrogen excretion [3], suggesting a rise in whole body protein turnover at luteal phase. In this context, amino acids that play a functional role in the preparation for successful pregnancy are of particular interest. While these amino acids may be essential at luteal phase, substantial utilization could reduce their availability. On the other hand, amino acids with specific immune regulatory functions could be kept low at luteal phase on purpose.

So far, only small-scale studies reported lower plasma arginine, citrulline, and ornithine concentrations at luteal compared to follicular phase [4,5]. While these changes have been postulated to be due to changes in progesterone levels [4,5], which show a sharp increase at luteal phase [6], direct evidence that these changes occur in response to elevated progesterone concentrations at luteal phase has not been provided. Several mechanisms, including transport, synthesis and recycling, have been proposed to be involved in maintaining arginine concentrations within a physiological range, which was reported as being 80–120 μmol/L [7], in the absence of published reference values.

Arginine is a conditionally essential amino acid for most mammals including humans [7], with the availability of citrulline being the limiting factor for *de novo* synthesis [8]. Arginine is a substrate for two competing enzymes, i.e., arginase, producing urea and ornithine [9], and nitric oxide (NO) synthase, producing NO and citrulline [10]. At luteal phase, high arginase expression was shown in endometrium [11], as was endothelial NO synthase (eNOS) expression in endometrium [12] and corpus luteum [13,14]. In female fertility, NO plays important roles in angiogenesis [15,16], endothelial function [17], endometrial receptivity and implantation [18].

A temporary suppression of the immune response at luteal phase of the menstrual cycle as well as during pregnancy is critical for materno-fetal tolerance [19–21]. Arginine exerts immune modulatory functions [22,23] by specifically up-regulating the expression of the T cell antigen receptor zeta chain (CD3ζ) [24,25], which in turn induces the TCR-to-nuclear factor kappa B (NF-κB) pathway [26], resulting in nuclear translocation of NF-κB [27], known as a central regulator of immune responses [28]. The activation of the NF-κB p65 subunit in peripheral blood mononuclear cells (PBMC) was reduced not only during pregnancy, but also already at luteal phase [20,29], along with a shift from T_H_1- to T_H_2-type cytokines [19–21], which has been implicated in the preparation of the endometrium for implantation [30,31].

Arginine, via ornithine, is also a precursor of polyamines [9], which play a critical role in the preparation of the endometrium for implantation, including endometrial cell proliferation [32]. Polyamines are synthesized from ornithine with ornithine decarboxylase as the rate-limiting enzyme, catalyzing the conversion of ornithine to putrescine. Putrescine, spermidine, and spermine are interconverted by highly regulated enzymatic reactions, including back-conversion via intermediate acetylated polyamines, catalyzed by acetyltransferases and oxidases [33,34].

Data on polyamine plasma concentrations in healthy subjects are generally limited in numbers of subjects and individual polyamines analyzed [35–39]. Only two of these studies addressed longitudinal changes across the menstrual cycle, which were restricted to spermidine and spermine in 4 women, showing individually different fluctuations [37], and spermine in 9 women, reaching peaks at late follicular phase [39], while putrescine and N-acetyl-putrescine have not been studied.

Considering available evidence as described above, we established the working concept of a possible progesterone-controlled regulatory node that is physiologically relevant in female reproduction, including (i) enhanced arginase and NO synthase activities, resulting in increased arginine utilization, (ii) reduced arginine-dependent T cell receptor CD3ζ expression, leading to reduced NF-κB p65 activation and, finally, to a shift from T_H_1- to T_H_2 immune response required for materno-fetal immune tolerance, and (iii) arginase-induced enhanced conversion of arginine to ornithine, enhancing the production of polyamines to ensure endometrial growth, all of which, in concert, are aimed at successful preparation of pregnancy.

Using a comprehensive approach, the present study focused on investigating, in healthy women at the plasma and PBMC level, (i) longitudinal changes in arginine and arginine-related amino acids, including ornithine and citrulline, and polyamines, including putrescine, spermidine, spermine and N-acetyl-putrescine, across a given menstrual cycle, as well as (ii) associations at luteal phase of circulating arginine and related amino acids and polyamines with progesterone and (iii) associations of changes thereof between follicular and luteal phase, and, last but not least, (iv) relations of arginine with NF-κB p65 activation, with the aim to provide data that could support the postulated physiological node at luteal phase of the menstrual cycle as described above.

## Materials and methods

### Study design and study subjects

Healthy women of the BIOCLAIMS cohort (1310 Austrian study subjects, 606 men and 704 women, established 2011–2014 within the European Commission’s Framework 7 collaborative project entitled “Biomarkers of Robustness of Metabolic Homeostasis for Nutrigenomics-derived Health Claims Made on Food”) not using hormonal contraceptives and with self-reported stable lengths of the individual menstrual cycles, were eligible for the study.

The individual time points of each investigation were determined for each woman based on basal body temperature records over at least two menstrual cycles prior to study entry, which helped to estimate the individual lengths of the follicular and luteal phases. The first day of menstrual bleeding was considered as the first day of the menstrual cycle (T0). Investigations were performed at T1, early follicular phase, day 5.8 ± 1.0; T2, late follicular phase, day 11.9 ± 1.8; T3, mid luteal phase, day 19.6 ± 2.4; and T4, late luteal phase, day 25.3 ± 2.1. The individual timing of blood drawings in the mornings of the 4 investigation days of each woman was kept the same in order to minimize the effects of possible circadian variability of the biomarkers under investigation.

Data on arginine concentrations of 292 healthy men of the VITAGE cohort (295 Austrian, Spanish and French study subjects, established 2000–2004 within the European Commission’s Framework 5 collaborative project entitled “Fat-soluble vitamin status and metabolism during ageing: functional and nutritional consequences”), included in reference ranges of four age groups published previously [40] were used for calculation of reference ranges (5^th^-95^th^ percentile) for comparison with the results obtained in women across the menstrual cycle.

The study was conducted in accordance with the Helsinki Declaration and the study protocol of the BIOCLAIMS study was approved by the Ethics Committees of the Medical University, Graz, Austria (reference number 23–306 ex 10/11), and the Karl-Franzens University, Graz, Austria (reference number GZ. 39/23/63 ex 2011/12), the study protocol of the VITAGE study by the Ethics Committees of the Medical University of Graz (reference number 10–149 ex 99/00), the Unité de Nutrition Humaine, Centre Auvergne Rhône-Alpes, Institut National de la Recherche Agronomique, Clermont-Ferrand, France (CCPPR-Auvergne, Clermont Ferrand, France, reference number PR-AU342), and Unitat de Recerca de Lipids I Arteriosclerosi, Facultat de Medicina, Universitat Rovira I Virgili, Tarragona, Spain (CEIC-HUSJR of the Universitat Rovira I Virgili, Reus, Spain, reference number 02-03-21_4NO7). Written informed consent was obtained from all women prior to study entry.

### Methods

Basal body temperature was determined by the study participants at rest in the morning under the tongue, using Cyclotest lady® ovulation thermometer (UEBE Medical, Wertheim, Germany), after receiving detailed instructions on how to perform the measurements and recordings on chart templates. Absence of pregnancy was confirmed prior to each investigation using the human chorionic gonadotropin (hCG) Pregnancy Rapid Test (Mexacare, Heidelberg, Germany). Blood was collected after an overnight fast, using Vacuette® (Greiner Bio-One, Frickenhausen, Germany) blood collection tubes and centrifuged immediately. Plasma and serum were obtained and aliquots stored at -80°C until analysis. Particular attention was paid to careful sample processing, given that elevated arginase activity in hemolytic blood samples resulted in reduced plasma arginine concentrations [41]. Samples of all 4 time points of individual study participants were analyzed within the same analytical run, given that analytical within-day variability is usually smaller compared to between-day variability.

Serum progesterone concentrations were determined using an ELISA from DiaMetra S.r.I. (Segrate, Italy). Plasma concentrations of arginine and citrulline were measured based on methods previously described by Roth [42] and Schwarz et al. [43] with modifications. After precipitation of plasma with perchloric acid following neutralization of the supernatant with sodium carbonate, the extracted amino acids were derivatized with o-phtalaldehyde and separated on an Ultrasphere 5 ODS column (250 x 4.6 mm, 5 μm, Hichrom, Reading, UK) with gradient elution. Quantification was performed based on ratios of fluorescence signals of the amino acid of interest to the internal standard norvaline. Within-day coefficients of variation (CVs) (at low/high concentrations) were 0.50/1.20% for arginine, and 0.70/1.10% for citrulline. Between-day CVs were 4.7/11.3% for arginine, and 4.0/4.3% for citrulline. Plasma concentrations of ornithine, putrescine, N-acetyl-putrescine, spermidine and spermine were determined by liquid chromatography-tandem mass spectrometry (LC/MS/MS) as described by Magnes et al. [35]. Two solid-phase extraction columns were online coupled to LC/MS/MS, minimizing the sample pretreatment to a single derivatization step. Within-day CVs were 1.7/4.2% for ornithine, 2.8/3.6% for putrescine, 14.2/1.0% for N-acetyl-putrescine, 6.0/5.0% for spermidine and 9.4/5.0% for spermine. Between-day CVs were 3.2/3.0% for ornithine, 3.5/4.1% for putrescine, 13.1/13.7% for N-acetyl-putrescine, 6.0/4.6% for spermidine and 15.5/19.2% for spermine.

For determination of the activation of the NF-*κ*B p65 subunit, blood was collected in BD Vacutainer® CPT^TM^ tubes (Becton, Dickinson and Company, Franklin Lakes, NJ, USA), and PBMC were isolated. Whole cell extracts were prepared using Active Motif^TM^ nuclear extract kit (Active Motif, Carlsbad, CA, USA), and activation of NF-κB p65 containing dimers was determined based on selective binding of activated dimers to their consensus binding sites in immobilized oligonucleotides, using the TransAM® NF-κB family kit (Active Motif, Carlsbad, CA, USA), as recently described in detail [29].

### Statistical analysis

Data were collected in Excel file and subjected to statistical analysis. Changes across the menstrual cycle were analyzed using repeated measures analysis of variance (ANOVA) and repeated measures ANOVA on ranks for normally and non-normally distributed data, respectively, along with all pairwise multiple comparison procedures (Holm-Sidak and Tukey tests, respectively). To study relations between dependent and independent variables at a given time point or changes between two time points, linear regression analysis was performed. For variables, which, based on current knowledge, could not be identified as dependent or independent variables, Spearman rank correlations were used. Reference ranges, using percentile intervals, were calculated from 292 healthy men for comparison. Comparisons between women at different time points and healthy men were performed using student *t* tests or Mann-Whitney Rank Sum tests, depending on data distribution. To adjust for multiple comparisons, *P <* 0.01 was considered significant. SigmaPlot version 13.0 (Systat Software, San Jose, CA, USA) was used for statistical analysis as well as for creating graphs. Box-and-whisker plots display the median, 1^st^ and 3^rd^ quartile (box), 1^st^ and 3^rd^ quartile plus/minus 1.5 times the interquartile range (whiskers), as well as the 5^th^ and 95^th^ percentiles (individual dots).

## Results

The 28 healthy women enrolled in the study were 34.2 ± 6.58 years old. There were no drop-outs and all women completed all 4 investigations. None of the women had positive pregnancy hCG testing at any of the 4 time points.

### Changes in progesterone and basal body temperature across the menstrual cycle (longitudinal approach)

**Fig 1:**
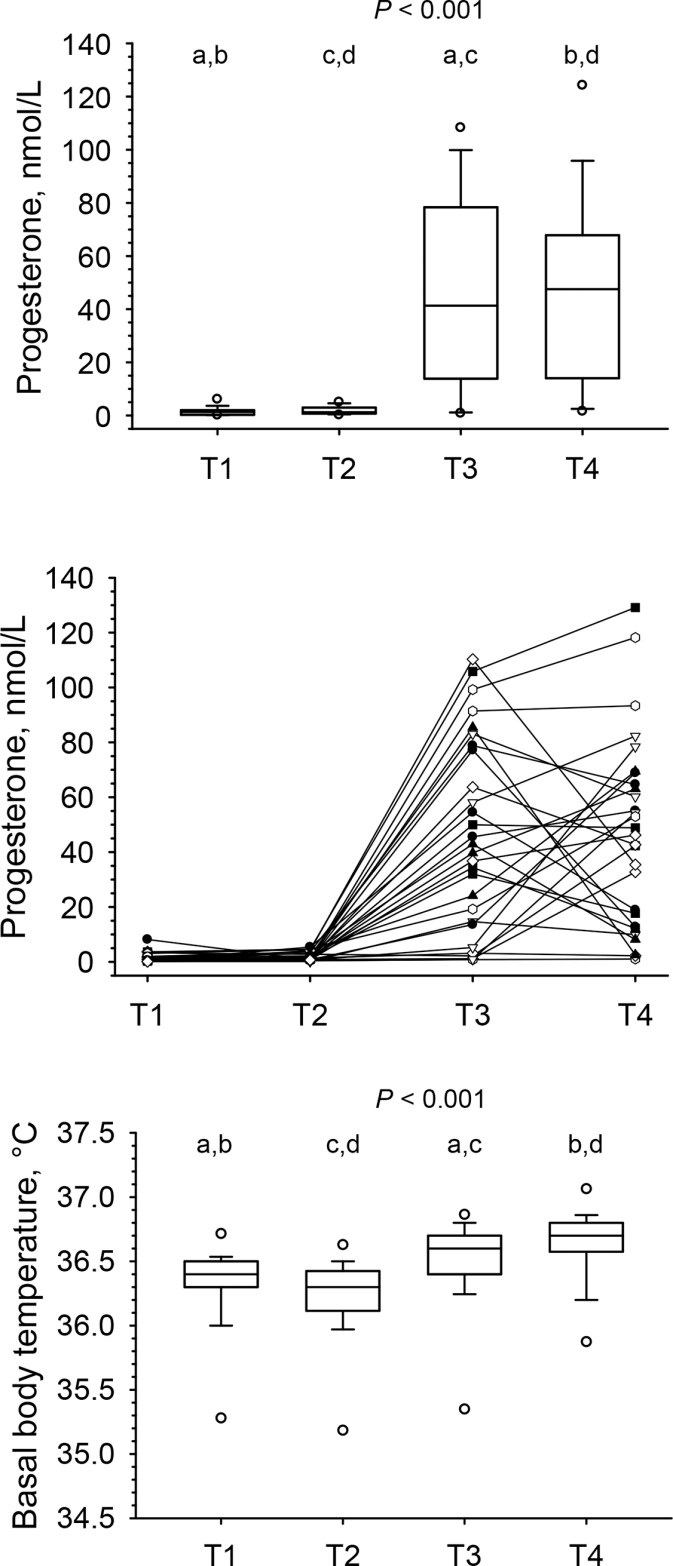
Serum progesterone concentrations, shown as box-and-whisker blots (upper panel) and as individual concentrations (intermediate panel), and basal body temperature (lower panel) at early (T1) and late (T2) follicular and mid (T3) and late (T4) luteal phase across a given menstrual cycle in 28 women. Results were obtained by repeated measures ANOVA (basal body temperature) and repeated measures ANOVA on ranks (serum progesterone) and all pairwise multiple comparison procedures (Holm-Sidak and Tukey test, respectively); same superscripts indicate significant differences (*P* < 0.01) between time points.

As shown in Fig 1, upper panel, serum progesterone concentrations increased strongly from T2 to T3 (*P* < 0.001), but did not change between T3 and T4. When looking at individual patterns (Fig 1, intermediate panel), progesterone concentrations increased in all but one women from T2 to T3, and 54% (*n* = 15) showed (further) increases from T3 to T4, while 46% (*n* = 13) showed decreases, resulting in a wide range of individual responses, which allowed for studying the effects on the different amino acids and polyamines of both increases and decreases in progesterone concentrations between T3 and T4. As expected, basal body temperature increased significantly from follicular to luteal phase (*P* < 0.001) (Fig 1, lower panel).

### Changes in amino acids across the menstrual cycle (longitudinal approach)

**Fig 2:**
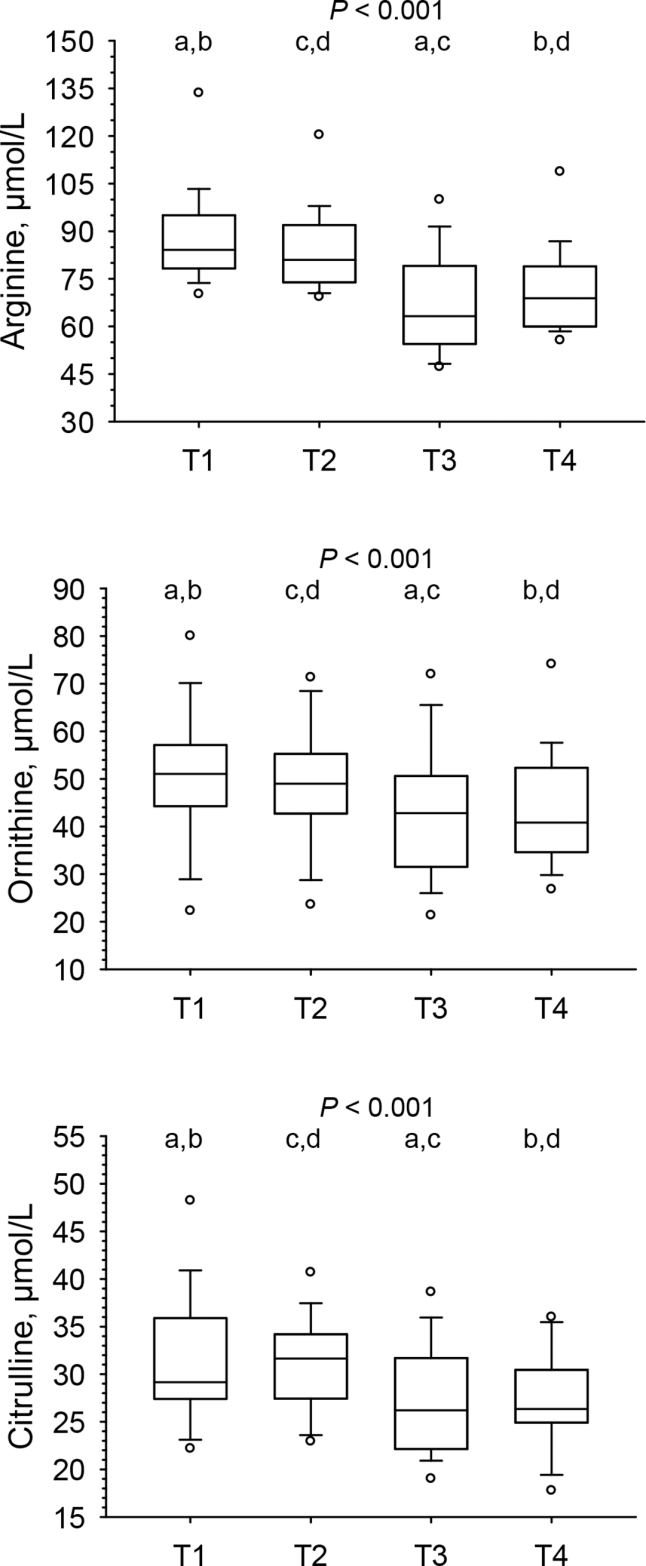
Plasma concentrations of arginine, ornithine and citrulline shown as box-and-whisker blots at early (T1) and late (T2) follicular and mid (T3) and late (T4) luteal phase across a given menstrual cycle in 28 women. Results were obtained by repeated measures ANOVA and Holm-Sidak all pairwise multiple comparison procedures; same superscripts indicate significant differences (*P* < 0.01) between time points.

Plasma concentrations of arginine, ornithine and citrulline decreased significantly from follicular to luteal phase (repeated measures ANOVA, *P* < 0.001; Holm-Sidak all pairwise multiple comparison procedures T1 vs. T3, T1 vs. T4, T2 vs. T3, and T2 vs. T4, all *P* < 0.01) (Fig 2). Changes between T2 and T3 (ΔT3-T2) were 17.1 μmol/L (19.8%) for arginine; 6.95 μmol/L (16.0%) for ornithine, and 3.78 μmol/L (12.1%) for citrulline. In Table 1, results are presented for all amino acids across the menstrual cycle. Compared to reference ranges of healthy males, plasma arginine concentrations were comparable at follicular phase (T1, T2), but significantly lower at luteal phase (T3, T4) (Table 2).

**Table 1:** Changes of variables across the menstrual cycle. Results are presented as mean ± SD of 28 women at 4 time points (T1-T4).

Variables	Follicular phase		Luteal phase		*P* value
	T1	T2	T3	T4	
*Amino acids*					
Arginine, μmol/L	87.9 ± 15.7^*a*.*b*^	84.2 ± 13.9^*c*.*d*^	67.1 ± 15.3^*a*.*c*^	70.9 ± 14.5^*b*.*d*^	<0.001
Citrulline, μmol/L	31.1 ± 6.75^*a*.*b*^	31.0 ± 5.02^*c*.*d*^	27.2 ± 5.66^*a*.*c*^	27.1 ± 5.12^*b*.*d*^	<0.001
Ornithine, μmol/L	47.5 ± 8.08^*a*.*b*^	43.5 ± 7.47^*c*.*d*^	36.6 ± 9.19^*a*.*c*^	36.5 ± 6.81^*b*.*d*^	<0.001
*Polyamines*					
Putrescine, μmol/L	0.084 ± 0.026	0.081 ± 0.027	0.082 ± 0.040	0.082 ± 0.025	0.498^#^
Spermidine, μmol/L	0.153 ± 0.043	0.139 ± 0.047	0.139 ± 0.040	0.139 ± 0.043	0.066^#^
Spermine, μmol/L	0.041 ± 0.018	0.044 ± 0.012	0.042 ± 0.015	0.044 ± 0.018	0.844^#^
N-acetyl-putrescine, μmol/L	0.066 ± 0.024	0.065 ± 0.021	0.067 ± 0.018	0.062 ± 0.014	0.738
*Ratios of ornithine to different polyamines*					
Ornithine to putrescine	608 ± 190 ^*a*^	585 ± 186	507 ± 190	475 ± 179 ^*a*^	0.001
Ornithine to spermidine	332 ± 95.1	353 ± 139	288 ± 115	294 ± 111	0.008^#^
Ornithine to spermine	1313 ± 497^*a*^	1074 ± 349	1035 ± 553	1028 ± 576^*a*^	0.005^#^
Ornithine to N-acetyl-putrescine	822 ± 361^*a*^	737 ± 273	588 ± 226^*a*^	622 ± 224	<0.001^#^
*Ratios between different polyamines*					
Putrescine to spermidine	0.575 ± 0.188	0.617 ± 0.194	0.606 ± 0.256	0.615 ± 0.183	0.758^#^
Spermidine to spermine	4.06 ± 1.33	3.22 ± 0.833	3.61 ± 1.20	3.51 ± 1.24	0.020
Putrescine to spermine	2.32 ± 1.08	1.94 ± 0.687	2.18 ± 1.32	2.15 ± 1.11	0.605^#^
Putrescine to N-acetyl-putrescine	1.43 ± 0.672	1.34 ± 0.551	1.31 ± 0.656	1.37 ± 0.525	0.832

^*a*,*b*,*c*,*d*^ Same superscript letters at different time points indicate significant differences (*P*<0.01); one-way repeated measures ANOVA and Holm-Sidak multiple comparison procedures

^#^ repeated measures ANOVA on ranks and Tukey multiple comparison procedure

**Table 2:** Comparison of plasma arginine concentrations (μmol/L) of women across the menstrual cycle with reference ranges of 292 healthy men.

	Healthy men *n = 292*	Women across the menstrual cycle *n = 28*			
		Early follicular phase, T1	Late follicular phase, T2	Mid luteal phase, T3	Late luteal phase, T4
Mean	84.7^a,b^	87.9	84.2	67.1^a^	70.9^b^
SD	16.3	15.7	13.9	15.3	14.5
5^th^ Percentile	58.8	70.2	69.3	47.3	55.7
10^th^ Percentile	64.5	73.7	70.5	48.2	58.4
25^th^ Percentile	72.7	78.3	73.9	54.5	59.9
50^th^ Percentile^c^	84.7	84.1	81.0	63.2	68.9
75^th^ Percentile	96.1	95.0	92.0	79.1	78.9
90^th^ Percentile	107	103	100	91.5	86.8
95P^th^ Percentile	112	134	120	100	109
Minimum	41.5	70.0	69.1	46.9	55.0
Maximum	126	146	138	102	126
Range	85.6	76.4	69.0	55.0	70.8

Differences between phases of the menstrual cycle and healthy men were analyzed using

t-tests (healthy men vs. mid luteal phase) or Mann-Whitney Rank Sum Test (healthy men vs. early follicular, late follicular and late luteal phase).

^a,b^ Same superscript letters indicate significant differences (*P*<0.01).

^c^ Median

### Regressions of amino acids on progesterone at T3

**Fig 3:**
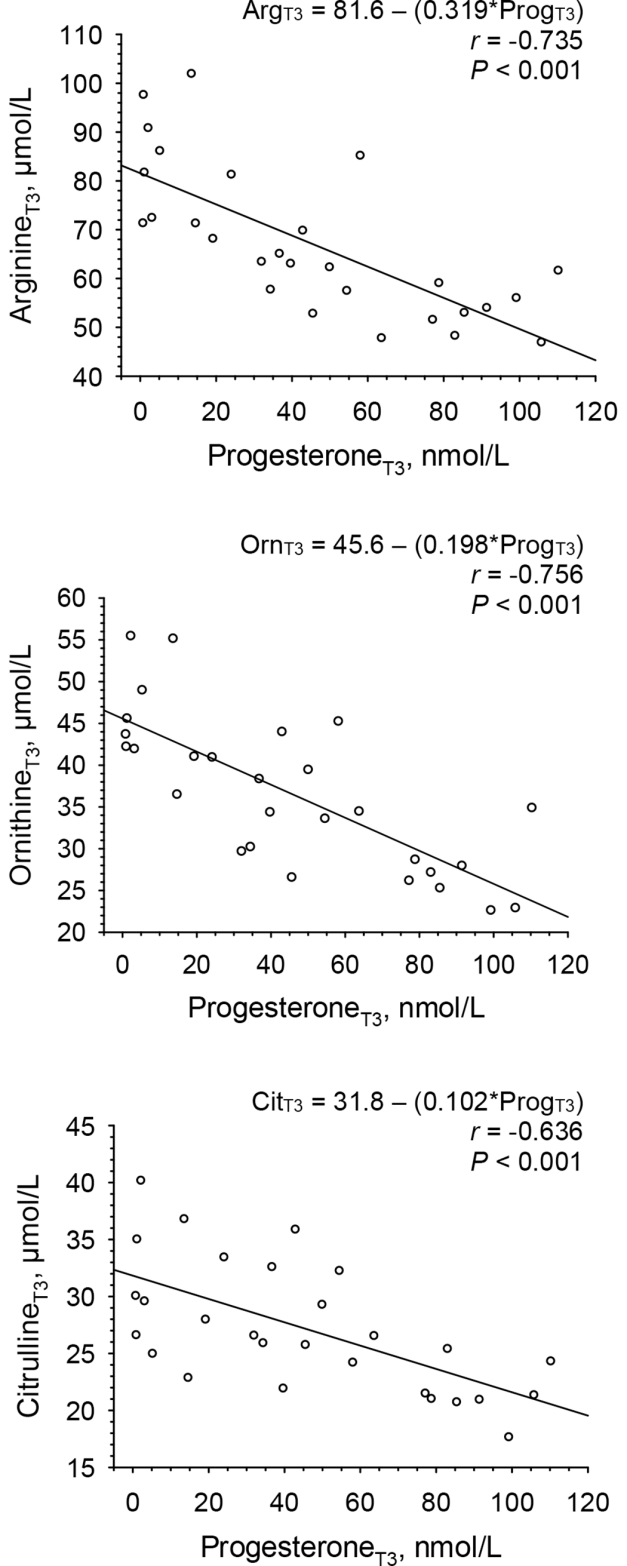
Linear regressions of plasma concentrations of arginine, ornithine and citrulline on serum progesterone concentrations at mid luteal phase (T3) of a given menstrual cycle in 28 women. Abbreviations: Arg, arginine; Cit, citrulline; Orn, ornithine; Prog, progesterone.

Regression coefficients of plasma concentrations of amino acids as the dependent (response) variables, on progesterone, as the independent (predictor) variable, are presented in Table 3. Inverse relations at T3, a time point with increased progesterone concentrations, were significant for arginine (*r* = -0.735), ornithine (*r* = -0.756), and citrulline (*r* = -0.636) (all *P* < 0.001). The coefficients (slopes) of the regression equations shown in Fig 3 were 0.319 μmol/nmol for arginine, 0.198 μmol/nmol for ornithine, and 0.102 μmol/nmol for citrulline.

**Table 3:** Regressions of amino acids (dependent variables) on progesterone (independent variables) at the 4 time points and regressions of changes (Δ) thereof between time points on Δ progesterone. Regression coefficients are presented for 28 women.

	T1	T2	T3	T4		Δ T2-T1	Δ T3-T2	Δ T4-T3
	Progesterone					Δ Progesterone		
Arginine	-0.150	0.004	-0.735^a^	-0.125	Δ Arginine	-0.206	-0.735^a^	-0.677^a^
Ornithine	0.033	0.011	-0.756^a^	-0.306	Δ Ornithine	-0.340	-0.693^a^	-0.705^a^
Citrulline	0.184	0.453	-0.636^a^	-0.414	Δ Citrulline	-0.353	-0.542^b^	-0.501^b^

^a^*P* < 0.001

^b^*P* < 0.01

### Regressions of changes in amino acids on changes in progesterone between T2 and T3 as well as between T3 and T4

Substantial between-women variability was observed in changes in progesterone concentrations between late follicular and mid luteal phase, including increases in all but one women, as well as between mid and late luteal phase, comprising in roughly 50% of women increases and decreases, respectively. This allowed for reliably studying effects of changes in progesterone concentrations (in both directions) on concentrations of amino acids and to compare results of linear regressions at T3 with those for changes between T2 and T3 as well as between T3 and T4.

In addition to regressions of amino acids and arginine-related derivatives at T3, linear regressions of longitudinal changes between T2 and T3 (ΔT3-T2) as well as between T3 and T4 (ΔT4-T3) in amino acid concentrations on longitudinal changes in progesterone concentrations were studied to further confirm relations between amino acids and arginine-related derivatives with progesterone. Results presented in Table 3 (panel on the right hand side), demonstrating significant regressions of changes in arginine, ornithine and citrulline concentrations between T2 and T3 (ΔT3-T2) as well as between T3 and T4 (ΔT4-T3) on changes in progesterone concentrations, confirmed results obtained at T3.

**Fig 4:**
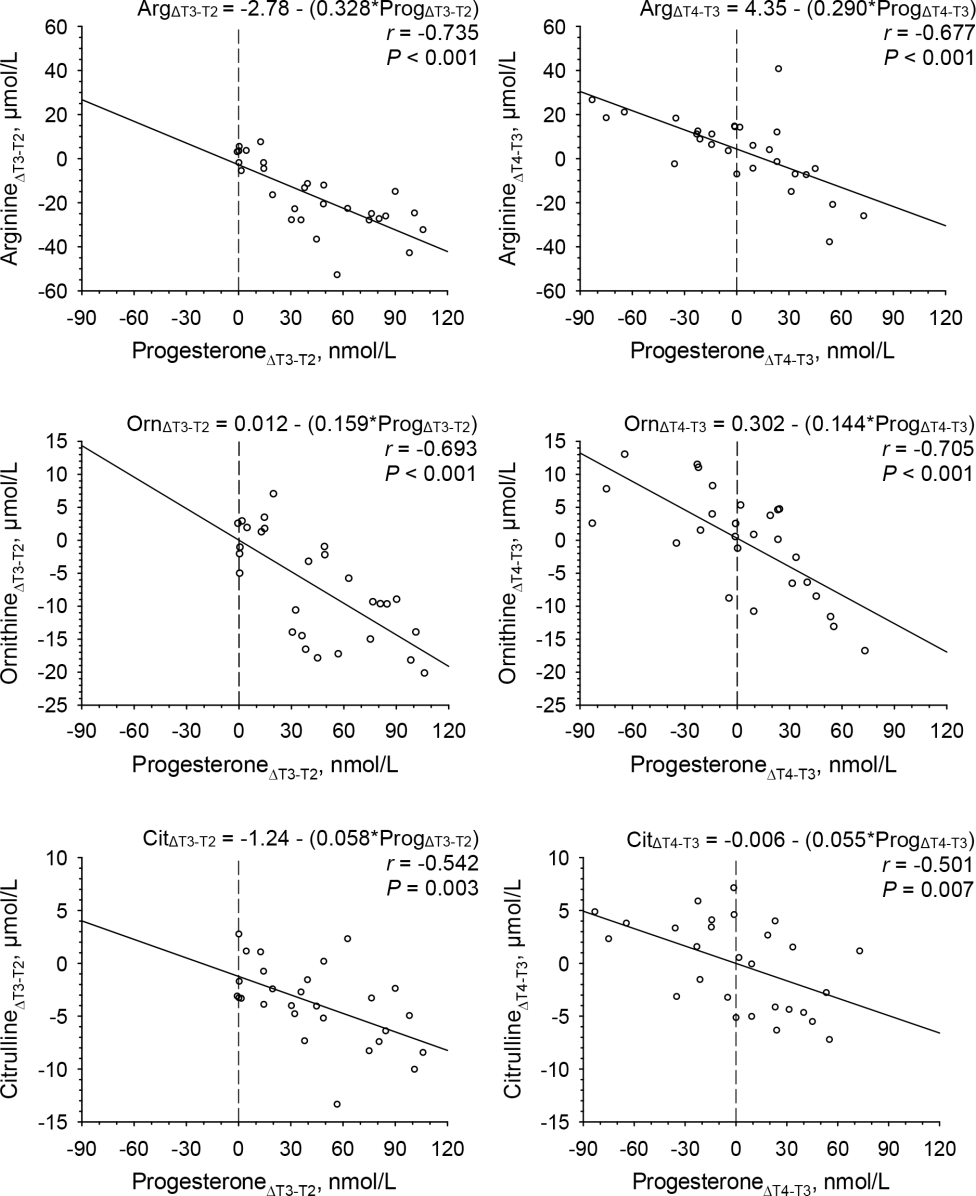
Linear regressions of changes in plasma concentrations of arginine, ornithine and citrulline on changes in serum progesterone concentrations between late follicular and mid luteal phase (ΔT3-T2) (panels on the left hand side) and between mid-luteal and late luteal (ΔT4-T3) phase (panels on the right hand side) of a given menstrual cycle in 28 women. Abbreviations: Arg, arginine; Cit, citrulline; Orn, ornithine; Prog, progesterone.

In Fig 4, details of linear regressions of changes in arginine, ornithine and citrulline concentrations on changes in progesterone concentrations between T2 and T3 (ΔT3-T2) as well as between T3 and T4 (ΔT4-T3) are presented. Between T2 and T3 (ΔT3-T2) (panel on the left hand side) progesterone concentrations increased in all but one women, while changes between T3 and T4 (ΔT4-T3) (panel on the right hand side) comprised both increases and decreases in progesterone concentrations. The slopes (equation coefficients) of the regressions of changes in arginine (0.328 versus 0.290 μmol/nmol), ornithine (0.159 versus 0.144 μmol/nmol), citrulline (0.058 versus 0.055 μmol/nmol) on changes in progesterone concentrations between T2 and T3 (ΔT3-T2) were comparable to those between T3 and T4 (ΔT4-T3). Furthermore, the slopes obtained for the regressions of changes in arginine on changes in progesterone concentrations between T2 and T3 as well as between T3 and T4 were comparable to the slope obtained at T3 (0.319 μmol/nmol), as shown in Fig 3.

### Correlations between amino acids

At T3, arginine concentrations were significantly related to those of ornithine (*P* < 0.001) and citrulline (*P* < 0.01). Ornithine concentrations were further significantly related to citrulline (*P* < 0.001) (Table 4, panel on the left hand side).

**Table 4:** Spearman rank correlations between arginine, ornithine and citrulline, at the 4 time points (T1-T4) as well as of changes (Δ) between time points. Correlation coefficients are presented for 28 women.

	T1	T2	T3	T4		Δ T2-T1	Δ T3-T2	Δ T4-T3
	Arginine					Δ Arginine		
Ornithine	0.436	0.519^b^	0.898^a^	0.675^a^	Δ Ornithine	0.363	0.816^a^	0.775^a^
Citrulline	0.035	0.345	0.587^b^	0.228	Δ Citrulline	0.211	0.702^a^	0.521^b^
	Ornithine					Δ Ornithine		
Citrulline	0.154	0.422	0.729^a^	0.417	Δ Citrulline	0.228	0.645^a^	0.603^a^

^a^*P* < 0.001

^b^*P* < 0.01

At T4, arginine concentrations were significantly related to ornithine concentrations (*P* < 0.001) (Table 4, panel on the left hand side).

### Correlations of changes in amino acids

As shown in Table 4 (panel on the right hand side), changes in arginine concentrations between T2 and T3 (ΔT3-T2) as well as between T3 and T4 (ΔT4-T3) were significantly related to changes in ornithine and citrulline concentrations (all *P* < 0.01). Changes in ornithine concentrations were further related to changes in citrulline concentrations, both between T2 and T3 (ΔT3-T2) (*P* < 0.001) and between T3 and T4 (ΔT4-T3) (*P* < 0.001) (Table 4, panel on the right hand side).

### Regressions of NF-κB p65 activation on arginine concentrations

**Fig 5:**
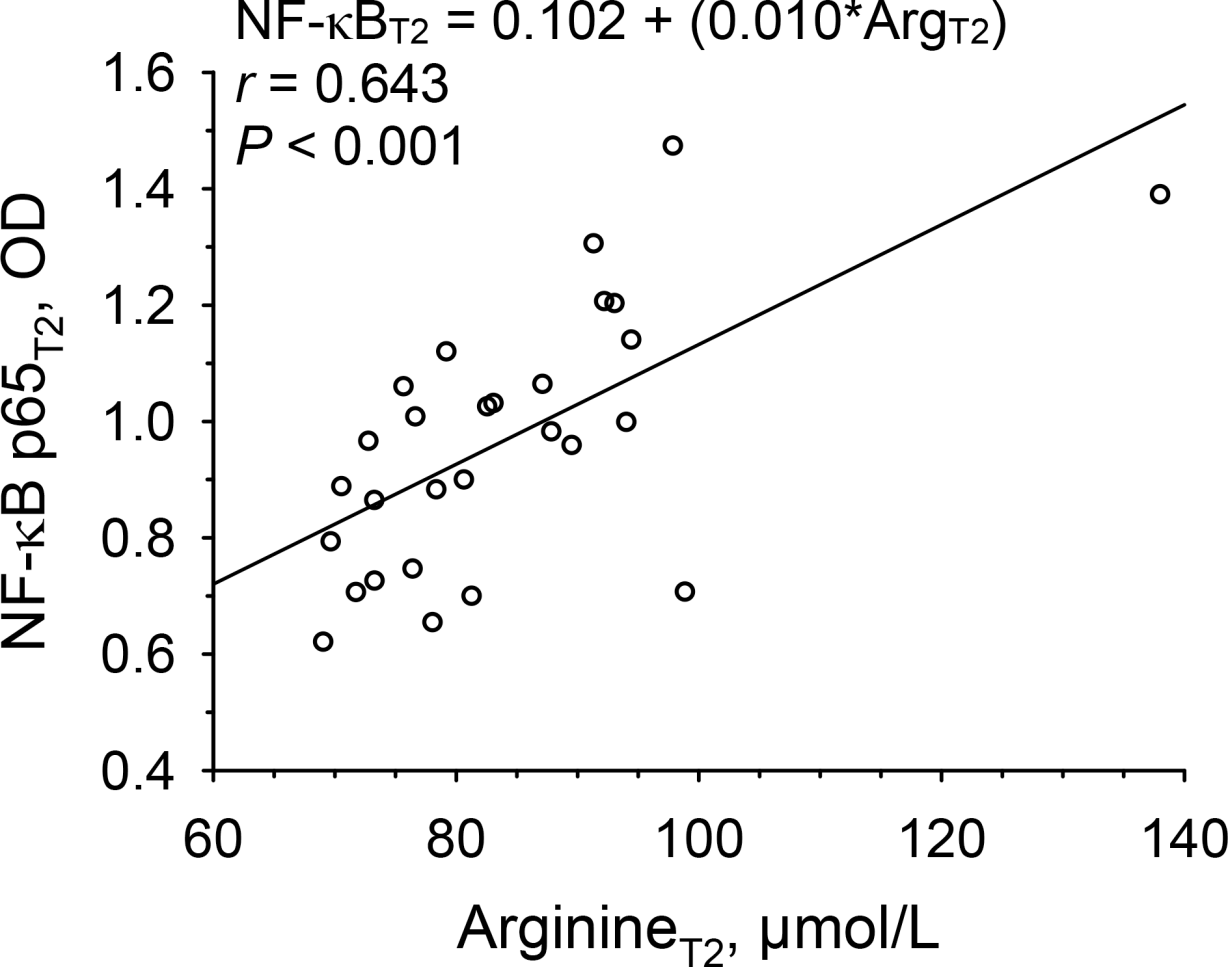
Linear regressions of activation of the NF-κB p65 subunit in PBMC on plasma arginine concentrations at late follicular phase (T2) of a given menstrual cycle in 28 women. Abbreviations: Arg, arginine, NF-κB, nuclear factor kappa B.

In Fig 5, a significant (*r* = 0.643, *P* < 0.001) positive regression of the activation of the NF-κB p65 subunit in PBMC on plasma arginine concentrations at T2 is presented. An increase in arginine concentrations by 1 μmol/L was associated with an increase in NF-κB p65 activation by 0.010 optical density (OD). Regressions were not significant at other time points. Changes between T2 and T3 (ΔT3-T2) in NF-κB p65 activation were not related to changes in arginine concentrations, nor were changes in NF-κB p65 activation related to changes in progesterone concentrations.

### Changes in polyamines and in ratios thereof across the menstrual cycle (longitudinal approach)

**Fig 6:**
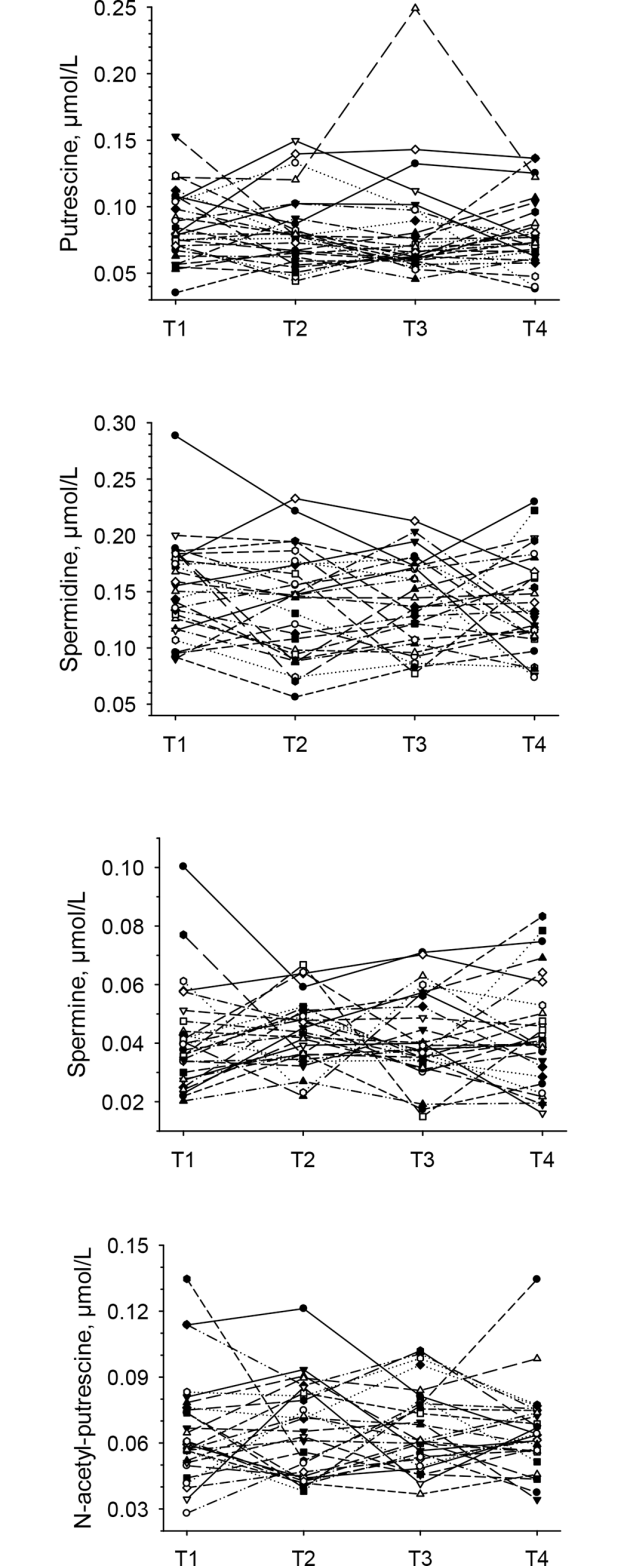
Plasma putrescine, spermidine, spermine and N-acetyl-putrescine shown as individual concentrations across a given menstrual cycle in 28 women.

**Fig 7:**
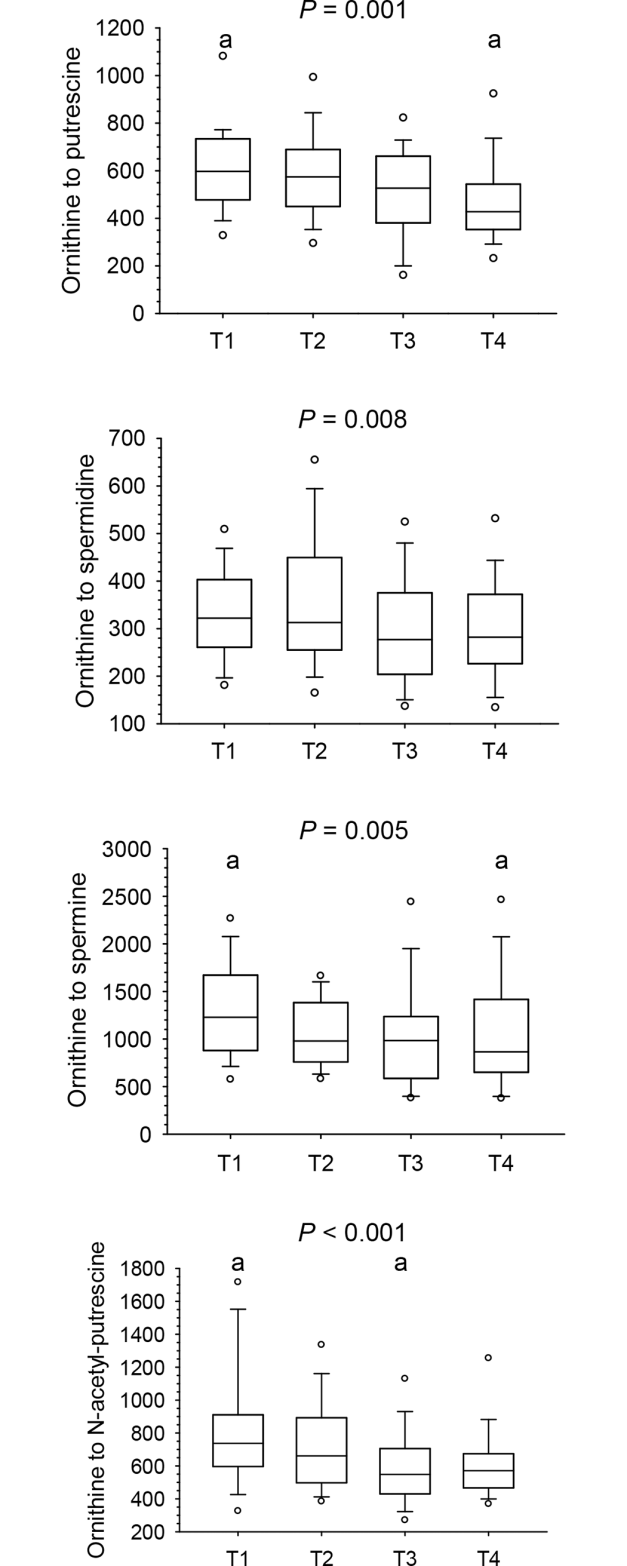
Ratios of ornithine to putrescine, spermidine, spermine and N-acetyl-putrescine shown as box-and-whisker blots at early (T1) and late (T2) follicular and mid (T3) and late (T4) luteal phase across a given menstrual cycle in 28 women. Results were obtained by repeated measures ANOVA and repeated measures ANOVA on ranks for normally and non-normally distributed data, respectively, along with all pairwise multiple comparison procedures (Holm-Sidak and Tukey tests, respectively); same superscripts indicate significant differences (*P* < 0.01) between time points.

Plasma concentrations are presented in Table 1. They were highest for spermidine, followed by putrescine, N-acetyl-putrescine and spermine (only about 30% of spermidine). Longitudinal changes in plasma putrescine, spermidine, spermine, and N-acetyl-putrescine across the menstrual cycle did not reach statistical significance in the entire study group (Table 1). When looking at individual women across a given menstrual cycle, substantial fluctuations, which differed between women and did not follow a specific pattern, became evident (Fig 6). Ratios of ornithine to putrescine, spermidine, spermine as well as to N-acetyl-putrescine, showed significant overall changes across the menstrual cycle (all *P* < 0.01), but post-hoc tests only reached significance (*P* < 0.01) for ratios of ornithine to putrescine, spermine as well as to N-acetyl-putrescine (Fig 7, Table 1). Ratios between different polyamines did not change across the menstrual cycle (Table 1).

### Regressions of polyamines and of ratios thereof on progesterone

**Fig 8:**
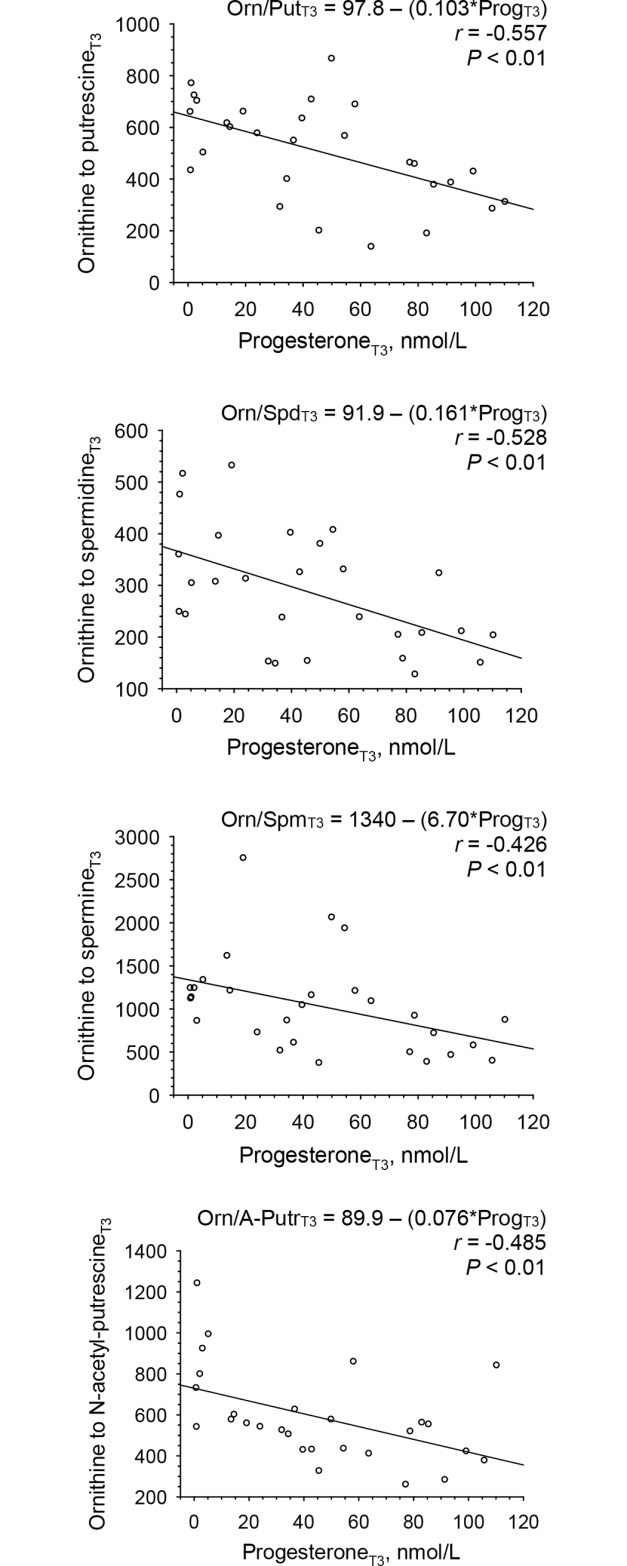
Linear regressions of plasma ratios of ornithine to putrescine, spermidine, spermine and N-acetyl-putrescine on serum progesterone concentrations at mid luteal phase (T3) of a given menstrual cycle in 28 women. Abbreviations: A-Putr, acetyl-putrescine; Orn, ornithine; Prog, progesterone; Put, putrescine; Spd, spermidine; Spm, spermine.

Ratios of ornithine to putrescine, spermidine, spermine as well as to N-acetyl-putrescine showed significant inverse relations (*P* < 0.01) with progesterone concentrations at T3 (Fig 8, Table 5, panel on the left hand side). Changes in ratios of ornithine to putrescine from T1 to T2 (ΔT2-T1) also showed a significant inverse relation with changes in progesterone concentrations from T1 to T2 (ΔT2-T1) (Table 5, panel on the right hand side). In contrast, changes in ratios of putrescine to spermidine were positively related to changes in progesterone concentrations (Table 5, panel on the right hand side).

**Table 5:** Regressions of polyamines (dependent variables) on progesterone (independent variable) at the 4 time points and regressions of changes (Δ) thereof between time points on Δ progesterone. Regression coefficients are presented for 28 women.

	T1	T2	T3	T4		Δ T2-T1	Δ T3-T2	Δ T4-T3
	Progesterone					Δ Progesterone		
Putrescine	0.135	0.024	0.142	0.080	Δ Putrescine	0.378	0.102	0.144
N-acetyl-putrescine	0.260	0.043	-0.013	0.097	Δ N-acetyl-putrescine	0.017	-0.172	-0.069
Spermidine	0.301	-0.075	0.063	0.022	Δ Spermidine	-0.151	0.153	0.186
Spermine	0.049	-0.058	0.203	0.339	Δ Spermine	0.155	0.225	0.351
Ornithine to putrescine	-0.115	-0.018	-0.557^b^	-0.141	Δ Ornithine to putrescine	-0.502^b^	-0.455	-0.345
Ornithine to spermidine	-0.365	0.210	-0.528^b^	-0.097	Δ Ornithine to spermidine	0.094	-0.450	-0.448
Ornithine to spermine	-0.159	0.119	-0.426^b^	-0.283	Δ Ornithine to spermine	-0.323	-0.416	-0.542^b^
Ornithine to N-acetyl-putrescine	-0.368	-0.050	-0.485^b^	-0.222	Δ Ornithine to N-acetyl-putrescine	-0.164	-0.210	-0.245
Putrescine to spermidine	-0.162	0.250	0.089	0.026	Δ Putrescine to spermidine	0.589^a^	-0.025	-0.085
Putrescine to spermine	-0.025	0.109	0.002	-0.236	Δ Putrescine to spermine	0.131	0.130	-0.319
Putrescine to N-acetyl-putrescine	-0.239	-0.022	0.209	-0.025	Δ Putrescine to N-acetyl-putrescine	0.131	0.218	0.225
Spermidine to spermine	0.172	-0.067	-0.111	-0.366	Δ Spermidine to spermine	-0.334	-0.106	-0.332

^a^*P* < 0.001

^b^*P* < 0.01

### Correlations between ornithine and polyamines and among polyamines

**Fig 9:**
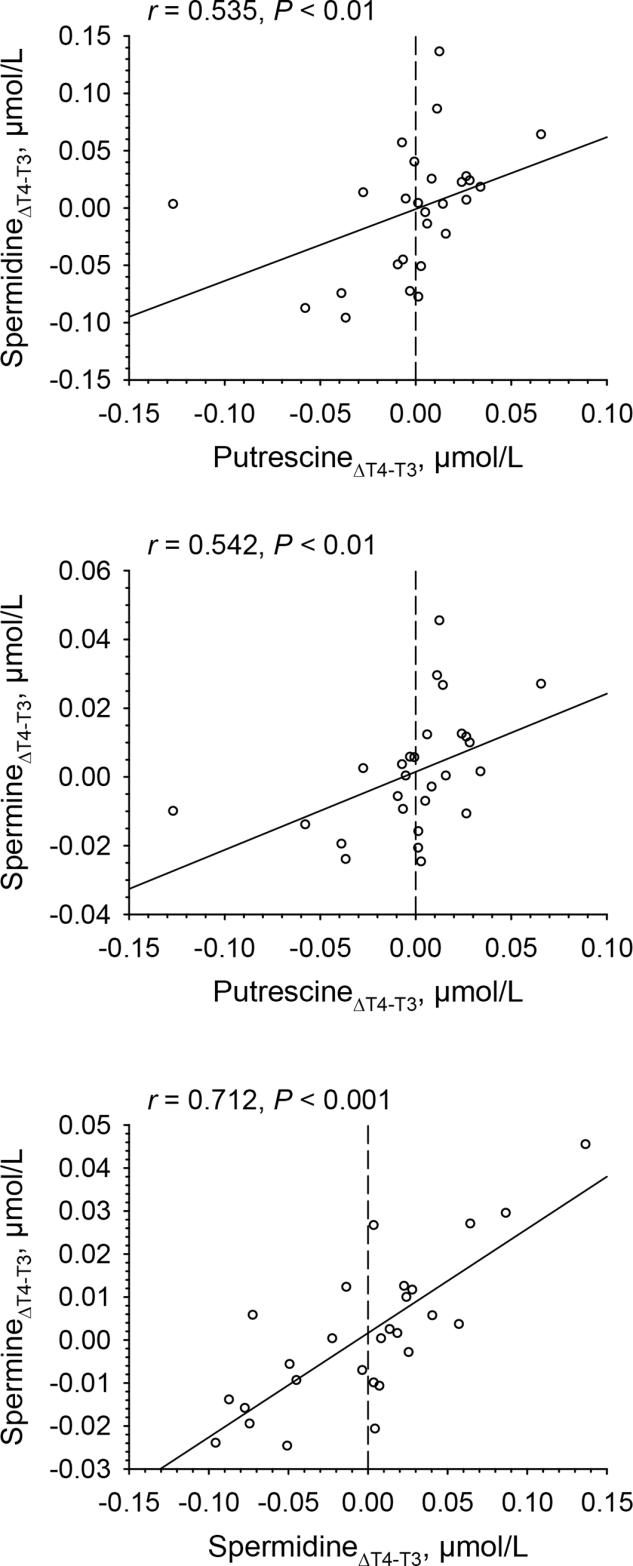
Spearman rank correlations between changes in plasma concentrations of putrescine and spermidine, putrescine and spermine as well as spermidine and spermine between mid and late luteal phase (ΔT4-T3) of a given menstrual cycle in 28 women.

Ornithine concentrations were not significantly related to putrescine, spermidine, spermine and N-acetyl-putrescine concentrations (Table 6, panel on the left hand side), nor were changes in ornithine concentrations related to changes in putrescine, spermidine, spermine and N-acetyl-putrescine (Table 6, panel on the right hand side). In contrast, spermidine concentrations were significantly related to putrescine concentrations at T2 (*P* < 0.01) and T3 (*P* < 0.001) (Table 6, panel on the left hand side), and spermine concentrations were significantly related to spermidine concentrations at T2 (*P* < 0.001) and T4 (*P* < 0.01) (Table 6, panel on the left hand side). Changes in putrescine concentrations between T3 and T4 (ΔT4-T3) were significantly related to changes in spermidine (*P* < 0.01) and spermine concentrations (*P* < 0.01), and changes in spermidine concentrations were significantly related to changes in spermine concentrations (*P* < 0.001) (Fig 9, Table 6, panel on the right hand side).

**Table 6:** Spearman rank correlations between ornithine and polyamines and among polyamines at the 4 time points (T1-T4) as well as of changes (Δ) between time points. Correlation coefficients are presented for 28 women.

	T1	T2	T3	T4		Δ T2-T1	Δ T3-T2	Δ T4-T3
	Ornithine					Δ Ornithine		
Putrescine	0.332	0.221	-0.007	0.078	Δ Putrescine	-0.088	0.053	-0.164
Spermidine	0.033	0.026	-0.087	-0.149	Δ Spermidine	0.311	-0.176	-0.218
Spermine	0.159	-0.137	-0.234	-0.274	Δ Spermine	-0.160	-0.032	-0.228
N-acetyl-putrescine	-0.157	-0.014	-0.011	-0.015	Δ N-acetyl-putrescine	-0.011	0.256	0.103
	Putrescine					Δ Putrescine		
Spermidine	0.243	0.558^b^	0.648^a^	0.378	Δ Spermidine	0.426	0.202	0.535^b^
Spermine	0.094	0.387	0.385	0.386	Δ Spermine	0.339	0.412	0.542^b^
N-acetyl-putrescine	0.158	0.322	0.002	0.175	Δ N-acetyl-putrescine	0.436	0.109	-0.412
	Spermidine					Δ Spermidine		
Spermine	0.435	0.642^a^	0.383	0.589^b^	Δ Spermine	0.409	0.259	0.712^a^

^a^*P* < 0.001

^b^*P* < 0.01

## Discussion

This study for the first time provides data obtained at the plasma and PBMC level at luteal phase of the menstrual cycle, supporting the proposed regulatory node, comprising arginine and related amino acids, progesterone and NF-κB p65, aimed at successful preparation of pregnancy by demonstrating, as the first important finding of the present study, progesterone-related effects on plasma concentrations of arginine, ornithine and citrulline of a given menstrual cycle, including (i) longitudinal changes with lower concentrations of the three amino acids along with higher progesterone concentrations at luteal phase, a finding that was confirmed by (ii) strong inverse relations of the three amino acids with progesterone at mid luteal phase, and strengthened by (iii) significant relations of changes in the three amino acid with changes in progesterone between late follicular and mid luteal as well as between mid and late luteal phase, and further highlighted by comparable slopes of the regression equations of (ii) and (iii).

Quantitatively, a 1 nmol/L increase in progesterone concentrations was related to a decrease in arginine concentrations of roughly 0.3 μmol/L, which was regarded as a strong indicator of a progesterone-controlled amino acid decline at luteal phase. Compared to late follicular phase, arginine at mid luteal phase was decreased by 19.8%, which is in line with previous small-scale studies showing changes of about 10–15% [4,5]. Compared to reference ranges of healthy males, using the same method in the same laboratory, plasma arginine concentrations were comparable at follicular phase, but significantly lower at luteal phase, reaching values below the 25^th^ percentile of healthy men in 86% (in 21 women at mid and 3 women at late luteal phase) and even below the 5^th^ percentile in 35.7% (in 10 women, all at mid luteal phase), suggesting that the luteal phase-specific arginine decline in the context of programmed preparation for successful pregnancy does not necessarily reach levels of deficiency, defined as being below the 5^th^ percentile of reference ranges.

Similar longitudinal changes in ornithine and citrulline concentrations suggest timed effects of the sharp increase in progesterone concentrations between late follicular and mid luteal phase on all three amino acids. For the first time, longitudinal changes were confirmed by strong inverse relations of all three amino acids with progesterone at mid luteal phase as well as between changes in the amino acids and progesterone between late follicular and mid luteal phase as well as between mid and late luteal phase. In contrast, at late luteal phase none of the three amino acids were significantly related to progesterone, probably because of absence of continuing physiological requirements in the absence of conception during the given menstrual cycle.

Regarding a mechanistic explanation of the findings, a number of luteal phase-specific morphological and functional changes shown in different studies may have contributed to arginine utilization, including increased expression of arginase [11] and eNOS [12] in endometrium as well as high expression of eNOS [13,14], NO-stimulated angiogenesis [15] and increased blood flow in corpus luteum [44]. Furthermore, a direct time- and dose-response effect of progesterone on eNOS expression was shown in endometrial cells [45] as was a relation between increased blood flow in corpus luteum and serum progesterone concentrations [44].

Differences in the magnitudes of declines in amino acid concentrations from late follicular to mid luteal phase, which were, in decreasing order, 19.8% for arginine, the main substrate for NO-synthase [46] and arginase [47]; 16.0% for ornithine, a precursor of polyamines [9]; 12.1% for citrulline, a substrate for *de novo* synthesis of arginine [8]; could be explained by different utilization and target levels at luteal phase according to their functional roles. Using stable-isotope labelling in healthy young men, 15% of plasma arginine was shown to be used by arginase for synthesis of ornithine and urea, while total arginine utilization for NO synthesis did not exceed 1.2% [48]. However, such data are not available in the present study, because arginase and NO synthase activity have not been determined.

Lower ornithine and citrulline concentrations at luteal phase may have occurred as a consequence of reduced arginine availability and/or further utilization of ornithine for polyamine synthesis on one hand and of citrulline for *de novo* synthesis of arginine to compensate for high demands at mid luteal phase on the other hand.

As the second important finding for the proposed role of arginine in modulating immune functions for successful pregnancy, plasma arginine concentrations for the first time were identified, by using regression analysis, as predictors of the activation of the NF-κB p65 subunit in PBMC at late follicular phase, linking arginine to the role of NF-κB p65 in the immune response shift required for materno-fetal immune tolerance. So far, NF-κB p65 activation in PBMC was shown to be decreased in pregnancy [20], as well as to decline from follicular to luteal phase of the menstrual cycle, while associations with arginine or progesterone concentrations have not been reported [29].

Regarding the underlying mechanisms of the findings of the present study, the arginine-mediated effect on NF-κB p65 activation could have been exerted through modulation of the T cell receptor CD3ζ expression [26], but CD3ζ expression was not determined in the present study. In trying to answer the question why similar relations were not observed at luteal phase, the strong effects of elevated progesterone on arginine concentrations, resulting in substantial utilization of arginine at luteal but not at follicular phase, could have jeopardized the regression of NF-κB p65 activation on arginine concentrations at luteal phase by selectively reducing arginine concentrations as the independent variable.

In contrast to arginine, changes in NF-κB p65 activation were not related to changes in progesterone concentrations in the present study. Reduced nuclear translocation of NF-κB p65 upon exposure of LPS-stimulated macrophages to 100 nmol/L progesterone suggested direct inhibitory effects of progesterone on NF-κB activation [49]; however, comparably high progesterone concentrations in plasma were only reached in three women of the present study. Interestingly, a study in healthy women showed higher NF-κB p65-DNA binding in the proliferative (at follicular phase) compared to the secretory (at luteal phase) endometrium [50], linking our findings in circulatory PBMC to evidence obtained in the endometrium.

Polyamine concentrations of the present study were comparable to those in 3 other studies including males and females [35,36,38], while the only 2 menstrual cycle studies reported 50- [37] and 2000-fold [39] higher spermidine and spermine concentrations. The analytical methods yielding the high concentrations were ion-exchange amino acid analyzer [37] and HPLC UV detection [39], while the other studies used solid phase extraction (SPE)-LC/MS/MS [35], LC-MS [36] and radioimmunoassay [38].

Even though changes in polyamine concentrations due to increased demands at luteal phase could have been expected, plasma putrescine, spermidine, spermine, and N-acetyl-putrescine concentrations did not vary significantly across the menstrual cycle in the present study. When looking at individual women, substantial changes in all 4 polyamines were observed, which is in line with another study reporting individually different fluctuations in spermidine and spermine concentrations [37]. Changes in all 4 polyamines between late follicular and mid luteal phase were not related to changes in progesterone.

In contrast, significant longitudinal changes in ratios of ornithine, the principal precursor of polyamines, to each of the 4 polyamines—with differences in ratios of ornithine to putrescine and ornithine to spermine between early follicular and late luteal phase and ratios of ornithine to N-acetyl-putrescine between early follicular and mid luteal phase -, as well as significant relations with progesterone at mid luteal phase could be attributed to the significant changes in and relations of ornithine with progesterone as the common numerator of the ratios.

The ratios of ornithine to putrescine and putrescine to spermidine could be regarded as proxies for the conversion of ornithine to putrescine and putrescine to spermidine, respectively. The inverse relation of changes between early and late follicular phase in progesterone concentrations with changes in ratios of ornithine to putrescine (i.e., with putrescine being the denominator) on one hand and positive relations of changes in progesterone with ratios of putrescine to spermidine (i.e., with putrescine being the numerator) on the other hand would suggest that progesterone exerts a putrescine-enhancing effect. However, progesterone levels at early and late follicular phase were very low and changes between early and late follicular phase were not significant. Moreover, such relations were not found in the presence of the pronounced increase in progesterone between late follicular and mid luteal phase, nor was an increase in putrescine in the luteal phase observed, when a pronounced increase in progesterone occured. Given that all 4 polyamines did not change significantly across the menstrual cycle and were neither related to progesterone concentrations at mid luteal phase, nor were changes in all 4 polyamines between late follicular and mid luteal phase related to changes in progesterone, these data suggest that generation of putrescine from ornithine and further conversions to spermidine and spermine as well as N-acetyl-putrescine are not controlled by progesterone.

In conclusion, the present study provides data, at the plasma and PBMC level, supporting a physiological regulatory node aimed at preparation of successful pregnancy, including robust data on progesterone-associated luteal phase-specific declines in circulating arginine and related amino acid concentrations, based on (i) longitudinal changes, (ii) strong inverse associations with progesterone concentrations, and (iii) significant associations between changes in arginine as well as arginine-related amino acids and changes in progesterone concentrations, and (iv) strong associations of declining arginine concentrations with declining NF-κB p65 activation, known to play a role in shifting the immune response towards materno-fetal tolerance, while generation of putrescine from ornithine and further to spermidine and spermine as well as N-acetyl-putrescine does not seem to be controlled by progesterone.

Based on the fact that blood continuously supplies tissues and organs, plasma and PBMC were used as a systemic resource for studying amino acids, progesterone, and NF-κB p65 activation in the present study. Interestingly, PBMC derived from non-pregnant women at luteal phase promoted progesterone production by human luteal cell cultures [51] and have thus been implicated in a systemic embryo-maternal cross-talk [52] and successfully used in *in vitro* fertilization [53], underpinning the physiological relevance of the proposed node at the circulatory level for preparation of successful pregnancy.

There are also limitations of this study, given that (i) associations do not establish cause-and-effect relationships, (ii) additional factors may also be important, as evidenced, for instance, by lack of association between NF-κB p65 activation and progesterone despite its relation to arginine, and (iii) absence of additional measurements in PBMC, for instance, of arginase and eNOS, that could have allowed to quantify arginine consumption and the proposed conversions between amino acids. Furthermore, relations between the circulatory and organ levels, including information on physiological responses, such as NF-kB activation in relation to changes in amino acids, in the corpus luteum and endometrium would be of interest. To fully characterize the regulatory node and further address the physiological significance of the data presented in female fertility, future investigations are needed.
